# Statistical Characteristics of Strong Earthquake Sequence in Northeastern Tibetan Plateau

**DOI:** 10.3390/e27020174

**Published:** 2025-02-06

**Authors:** Ying Wang, Rui Wang, Peng Han, Tao Zhao, Miao Miao, Lina Su, Zhaodi Jin, Jiancang Zhuang

**Affiliations:** 1 Shaanxi Earthquake Agency, Xi’an 710068, China; wy200543@163.com (Y.W.); zhaotaowy@163.com (T.Z.); sulinawhu@163.com (L.S.); jinxiaoyu991150@163.com (Z.J.); 2 Department of Earth and Space Science, Southern University of Science and Technology, Shenzhen 518055, China; hanp@sustech.edu.cn (P.H.); miaom@sustech.edu.cn (M.M.); 3 Key Laboratory of Earthquake Forecasting and Risk Assessment, Ministry of Emergency Management, Southern University of Science and Technology, Shenzhen 518055, China; 4 Guangdong Provincial Key Laboratory of Geophysical High-Resolution Imaging Technology, Southern University of Science and Technology, Shenzhen 518055, China; 5 China Nuclear Power Engineering Co., Ltd., Shenzhen 518172, China; 6 Institute of Statistical Mathematics, Tokyo 190-8562, Japan; zhuangjc@ism.ac.jp

**Keywords:** earthquake sequence, statistical analysis, ETAS model, *b* value, bi-scale transformation

## Abstract

As the forefront of inland extension on the Indian plate, the northeastern Tibetan Plateau, marked by low strain rates and high stress levels, is one of the regions with the highest seismic risk. Analyzing seismicity through statistical methods holds significant scientific value for understanding tectonic conditions and assessing earthquake risk. However, seismic monitoring capacity in this region remains limited, and earthquake frequency is low, complicating efforts to improve earthquake catalogs through enhanced identification and localization techniques. Bi-scale empirical probability integral transformation (BEPIT), a statistical method, can address these data gaps by supplementing missing events shortly after moderate to large earthquakes, resulting in a more reliable statistical data set. In this study, we analyzed six earthquake sequences with magnitudes of *M*_S_ ≥ 6.0 that occurred in northeastern Tibet since 2009, following the upgrade of the regional seismic network. Using BEPIT, we supplemented short-term missing aftershocks in these sequences, creating a more complete earthquake catalog. ETAS model parameters and *b* values for these sequences were then estimated using maximum likelihood methods to analyze parameter variability across sequences. The findings indicate that the *b* value is low, reflecting relatively high regional stress. The background seismicity rate is very low, with most mainshocks in these sequences being background events rather than foreshock-driven events. The p-parameter of the ETAS model is high, indicating that aftershocks decay relatively quickly, while the α-parameter is also elevated, suggesting that aftershocks are predominantly induced by the mainshock. These conditions suggest that earthquake prediction in this region is challenging through seismicity analysis alone, and alternative approaches integrating non-seismic data, such as electromagnetic and fluid monitoring, may offer more viable solutions. This study provides valuable insights into earthquake forecasting in the northeastern Tibetan Plateau.

## 1. Introduction

Earthquakes are among the most devastating natural disasters worldwide. The largest and most destructive seismic events typically occur at plate boundaries. Notable examples include the 1960 *M*_W_9.5 Valdivia earthquake in Chile, the 2010 *M*_W_8.8 Maule earthquake in Chile, the 2011 *M*_W_9.0 Tohoku earthquake in Japan, and the 2007 *M*_W_8.5 Sumatra earthquake, among others [1,2,3,4,5]. However, accurate earthquake prediction remains an unsolved challenge. To advance this field, many researchers have concentrated on seismically active regions, including Sichuan-Yunnan in China, Japan, California in the United States, and New Zealand, and conducted extensive observations. Utilizing a wealth of data from these regions, researchers have developed various statistical and physical methods to identify patterns of seismic activity, which has led to improvements in prediction efficiency. Examples of such methods include the *b* value, the Epidemic-Type Aftershock Sequence (ETAS) model, as well as methods for earthquake identification and localization [6,7,8,9,10,11,12]. However, these methods are not applicable in certain regions, making earthquake prediction in these areas particularly challenging.

The Tibetan Plateau is a region of particular geological significance, characterized by its highly complex geological structure. The northeastern section of the plateau represents the frontal region, extending into the mainland [13,14]. Due to the extension of the plateau, stress continuously accumulates; however, the strain rate is approximately 2 mm/year [15], significantly lower than that observed in subduction zones, and the earthquake frequency is relatively low [16]. This discrepancy results in a high level of stress within the region. The high stress levels combined with the complex geological conditions make this region one of the areas with the highest earthquake hazard, contributing to its complex seismicity. Historically, the region has experienced numerous large earthquakes, one of the most notable being the 1920 *M*8^1^/_2_ Haiyuan earthquake, renowned as one of the most destructive events in seismic history. In recent years, significant seismic events have occurred, including the *M*_S_7.4 earthquake in Maduo in 2021; the *M*_S_6.9 earthquake in Menyuan, Qinghai Province, in 2022; and the Maerkang *M*_S_6.0 earthquake swarm in Sichuan Province in 2022. These events suggest increased seismic activity in the northwestern part of the Qinghai–Tibet Plateau, highlighting the region’s substantial earthquake hazard and drawing considerable attention from seismologists. A clear understanding of seismicity characteristics in this area is essential for effective earthquake prevention and disaster response efforts. With the comprehensive upgrade of the seismic network since 2009, earthquake monitoring capabilities have significantly improved, providing an invaluable opportunity to investigate seismicity characteristics through statistical approaches.

The ETAS model is a widely used tool for simulating the temporal evolution of seismicity rates. As one of the most realistic time series models, the ETAS model has been employed by researchers to analyze earthquake sequences across different regions, yielding insights into the characteristics of seismic activity. In China, some scholars [17,18] have utilized the ETAS model to investigate the distribution characteristics of earthquake sequence parameters. As a method based on statistics, the ETAS model relies heavily on earthquake catalogs as its data source. However, the effectiveness of earthquake monitoring can decline significantly following a major earthquake, both within the affected area and potentially in surrounding regions, due to the difficulty of detecting smaller earthquakes amidst the occurrence of numerous moderate to large earthquakes [19,20]. Consequently, a significant amount of post-earthquake data remain unrecorded, resulting in an increase in the number of incomplete magnitude readings. This scarcity can severely undermine the accuracy and stability of parameters fitting within the ETAS model. One relatively straightforward approach to mitigate this issue is to select a higher threshold magnitude; however, a low number of recorded earthquakes can also lead to unstable results [21,22]. Alternative methods aim to address catalog completeness. The template matching technique and other earthquake recognition methods are frequently employed to identify smaller earthquakes, but they require an extensive earthquake observation system [23,24,25,26,27]. The complex terrain of the northeastern Tibetan Plateau complicates the establishment of a dense seismic network, making the application of template matching in this region challenging. The bi-scale empirical probability integral transformation (BEPIT) method proposed by Zhuang et al. [28,29] can be applied to seismic sequences with sparse seismic stations and weak early monitoring ability based on the statistical law of aftershock attenuation. This method has been proven to help estimate stable attenuation parameters and obtain parameter information of early post-earthquake aftershock sequences [30,31]. Consequently, this method is employed in the present investigation to replenish the catalog of earthquake events immediately following the main shock.

In this study, we investigate the statistical characteristics of strong earthquake sequences (*M*_S_ ≥ 6.0) in the northeastern Tibetan Plateau since 2009. Prior to analysis, we apply a seismic event replenishment technique based on statistical seismology to address the low seismicity rate and the incompleteness of the earthquake catalog following major earthquakes. Our analysis focuses on the seismic sequence parameters of these events, utilizing the ETAS model and the *b* value to elucidate the characteristics of seismicity in the region. Subsequently, we discuss the implications and potential applications of these findings in earthquake forecasting. This study seeks to contribute meaningful insights toward improving earthquake forecasting in the northeastern Tibetan Plateau.

## 2. Materials and Methods

### 2.1. Study Area and Data Selection

**Figure 1:**
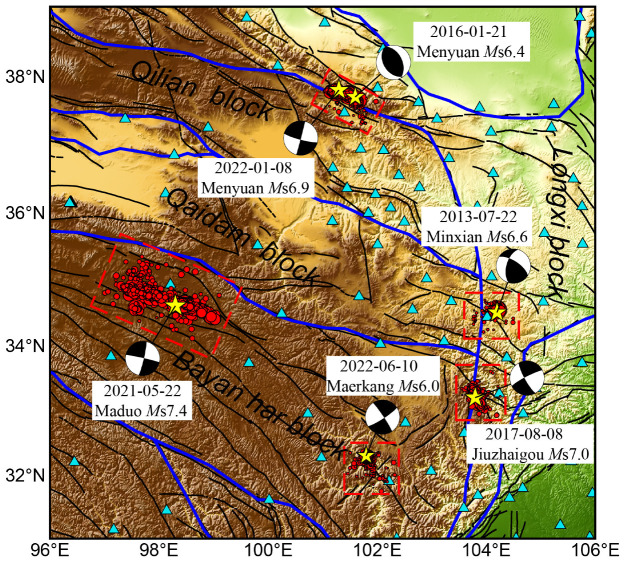
The spatial distribution of six earthquake sequences with a mainshock of *M*_S_ ≥ 6.0 in the northeastern Tibetan Plateau since 2010. The seismic events are represented by red dots, scaled according to their magnitudes. The mainshocks of each sequence are distinguished by yellow five-pointed stars, and the individual focal mechanisms of the mainshocks are marked. Tectonic blocks are delineated by solid blue lines, while tectonic fractures are depicted by solid black lines. Additionally, observation stations are denoted by light blue triangles in the diagram. The focal mechanism results were obtained from the Global CMT Catalog (www.globalcmt.org).

Six earthquake sequences with *M*_S_ ≥ 6.0 have occurred on the northeastern Tibetan Plateau since 2009 and were selected for investigation. In the study region, seismic station coverage is sparse; as shown in Figure 1, there are four or fewer seismic stations within 100 km of each mainshock epicenter. Figure 1 displays the locations of these seismic events, which include the following: the 2013 *M*_S_6.6 earthquake sequence in Minxian, Gansu Province; the 2016 *M*_S_6.4 earthquake sequence in Menyuan, Qinghai Province; the 2017 *M*_S_7.0 earthquake sequence in Jiuzhaigou, Sichuan Province; the 2021 *M*_S_7.4 earthquake sequence in Maduo, Qinghai Province; the 2022 *M*_S_6.9 earthquake sequence in Menyuan, Qinghai Province; and the 2022 *M*_S_6.0 earthquake cluster in Maerkang, Sichuan Province. The earthquake data utilized in this study were obtained from the China Earthquake Networks Center (CENC). Only events with *M*_L_ ≥ 0.0 are included in the data set. In selecting earthquake sequences, priority is given to intensive aftershock areas that develop within one month following each main event. The data retained for analysis also include the period preceding the main earthquake, enabling an examination of background seismic activity based on the level of seismicity and magnitude of the main event within the selected region.

Complete seismic samples are the basis of reliable results. The complete magnitude of completeness (*M*_C_) is the smallest magnitude at which an earthquake can be completely monitored. It is an index to evaluate the quality of an earthquake catalog. In this study, the maximum curvature (MAXC) method is applied to estimate the temporal *M*_C_ of the sequence catalog [32]. This approach aids in estimating a new empirical distribution function based on fully recorded seismic data. However, previous studies indicate that the complete magnitude calculated by the MAXC method tends to be relatively low [33]. Therefore, in this study, we adjusted the MAXC result by adding 0.2 to define the complete magnitude. Using a moving time window, we conducted a temporal *M*_C_. The higher *M*_C_ before and after the main shock are set to be the thresholds of magnitude for replenishing to ensure the completeness of the earthquake catalog for most of the time except for immediately after the mainshock. The *M*_C_ after supplementing is shown in Table 1.

### 2.2. Seismic Event Supplement Technique Based on Statistical Seismology

Randomly occurring seismic events are commonly regarded as marking point processes [34,35,36,37]. Based on this framework, Zhuang et al. [28,29] proposed a novel algorithm, the BEPIT, employing a two-scale transformation technique. This algorithm was designed to supplement missing seismic events in incomplete records.

Suppose $N=\left\{\left(t_{i},\;m_{i}\right):i=1,\;2,\;\ldots ,\;n\right\}$ is a temporal marked point process in a time–magnitude domain [0,T]×M, where *M* is the space of marks. Using the following BEPIT, the empirical distribution function of time and magnitude is defined:

(1) $$\Gamma_{N}:\left[0,T\right]\times M\;\to \;\left[0,1\right]\times [0,1]$$

(2) $$\left(t,m\right)\;\to \;\left(t^{\prime },m^{\prime }\right)=\left\{\frac{1}{n}\sum_{j=1}^{n}I\left(t_{j}<t\right),\;\frac{1}{n}\sum_{j=1}^{m}I\left(m_{j}<m\right)\right\}$$

where *I* is a logical function. It is defined as *I*(*x*) = 1 if *x* is true or as *I*(*x*) = 0 if *x* is false. 

BEPIT is used to convert the non-uniform mode generated by the actual point process into a uniform mode, and then the missing aftershock events are filled in. The method comprises the following five main steps (for a detailed description of the method, see Zhuang et al. [28,29]):

**Step 1.** Seismic events are transformed to a [0, 1] × [0, 1] range using the BEPIT point processing technique, and the approximate time–magnitude range is established for missing events, S.

**Step 2.** Based on the seismic events within the time–magnitude range that should be fully observed, a new empirical distribution function is obtained by searching for the uniform distribution of events through an iterative process and adjusting the missing event region, S.

**Step 3.** Based on the empirical distribution function of the previous step, independent and uniformly distributed events are generated in the missing event region.

**Step 4.** Since the missing region will also contain some real seismic events, the supplementary data set needs to be adjusted. Duplicate events in the synthetic data set can be avoided by deleting the most recent supplementary data point for each real event.

**Step 5.** By means of linear interpolation, the magnitude of each supplementary event is assigned based on the observed relationship between magnitude and time.

### 2.3. ETAS Model

Ogata [38] extended the Omori–Utsu law by applying the Hawkes point process, positing that aftershocks exhibit self-similarity and that each earthquake has the potential to generate aftershocks. This leads to a cascading series of direct and indirect aftershocks following a main event. He introduced the ETAS model, characterized by a branching point process structure.

The intensity function of the seismic series $\left\{\left(t_{i},\;\;M_{i}\right);i=1,2,\ldots ,N\right\}$ in the subsequent observation period [0, T] with parameters [*μ*, *A*, *c*, *α*, *p*] can be expressed as follows [34,38]:

(3) $$\lambda \left(t\right)=\mu +\sum_{t_{i}<t}Ae^{\alpha (M_{i}-M_{0})}\frac{p-1}{c}{\left(\frac{t-t_{i}}{c}+1\right)}^{-p}\;\;M_{i}>M_{0}$$

where *t* represents the elapsed time after the initial zero time. *M*_i_ and *t*_i_ refer to the magnitude and onset time of the *i*th event, respectively. *M*_0_ is the reference magnitude, which is usually taken as the cutoff magnitude that is equal to or greater than the complete magnitude *M*_C_. The background seismicity rate is denoted by *μ* and is caused by the loading of regional stress, but it is usually negligible when compared to the aftershock sequence. The *p* value indicates how quickly the sequence decays; the larger this value is, the faster the decay, and vice versa. The length of time during which the frequency of aftershocks reaches its peak after the main earthquake is represented by *c*. *A* represents the average number of earthquakes triggered by an earthquake of magnitude *M*_C_. *α* indicates the ability to trigger aftershocks. When it comes to swarm-type sequences, *α* is generally less than 1; if there is no obvious stimulated aftershock in the earthquake sequence, *α* is generally greater than 1 [39].

The ETAS model parameters are estimated using the maximum likelihood method, with the likelihood function *L* taking the form in [*S*, *T*] in the fitting time range.

(4) $$lnL=\sum_{i:S\leq t_{i}<T}ln\lambda \left(t_{i}\right)-\int_{S}^{T}\lambda \left(t\right)dt$$

We perform the maximum likelihood estimation of parameters [*μ*, *A*, *c*, *α*, *p*] by bringing Equation (3) into Equation (4) and maximizing Equation (4).

### 2.4. b Value

The Gutenberg–Richter (G-R) law indicates the frequency–magnitude distribution of earthquakes [40,41] as follows:

(5) $$lgN=a-bM\;(M\geq M_{C})$$

where *N* is the number of earthquakes with a magnitude greater than or equal to *M*, *a* is the earthquake productivity, and *b* is the slope indicating the relative proportion of large and small earthquakes. Research has demonstrated that the *b* value is inversely related to the underground stress level [42,43,44,45,46,47]. As such, the *b* value serves as an indicator of geological structure and stress conditions and is widely used in earthquake forecasting and related research [10,48,49].

## 3. Results

### Supplemental Data on Missing Earthquakes

To address the shortage of short-term aftershock data following an earthquake, we employed the BEPIT method. Using a moving time window, we conducted a temporal *M*c analysis for each of the six sequences, and the maximum *M*c values are summarized; the resulting *M*_C_ values are shown in Table 1. In the immediate aftermath of the main earthquake, some aftershock signals were obscured by large-amplitude tail waves [50,51], leading to a temporary increase in *M*_C_. As monitoring capabilities in the aftershock region gradually improved, *M*_C_ began to decrease. However, with the reduction in mobile observation stations, *M*_C_ gradually increased again, eventually stabilizing at levels similar to those observed prior to the main event.

**Figure 2:**
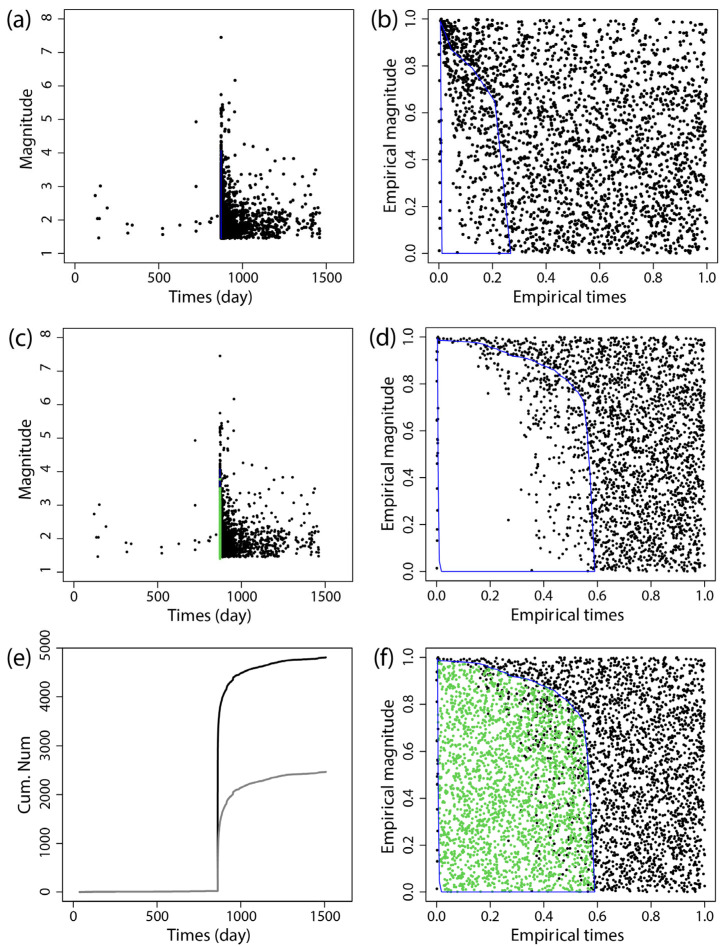
The supplementary results of the aftershock data of the 2021 *M*_S_7.4 Maduo earthquake obtained by using BEPIT. (**a**) shows the magnitude and occurrence time of actual seismic events and missing event ranges; (**b**) shows the empirical distribution of magnitude and the event occurrence time after the double-scale transformation technique (Step 1); (**c**) shows the magnitude and occurrence time of actual and supplementary seismic events (Step 5); (**d**) shows the empirical distribution of magnitude and the event occurrence time outside of the missing event range after the double-scale transformation technique (Steps 2 and 3); (**e**) shows the cumulative number of earthquakes in the original data set (gray curve) and the supplementary data set (black curve); and (**f**) shows the empirical distribution of the magnitude and timing of actual and supplementary seismic events (Step 4). The blue polygon on (**a**,**b**,**d**,**f**) is the region where the missing event is located, and its corresponding mapping and the green dots on (**c**,**f**) are the supplementary events. The *x*-axis and *y*-axis in (**b**,**d**,**f**) are the empirical time and empirical magnitude transformed from time and magnitude by BEPIT.

**Figure 3:**
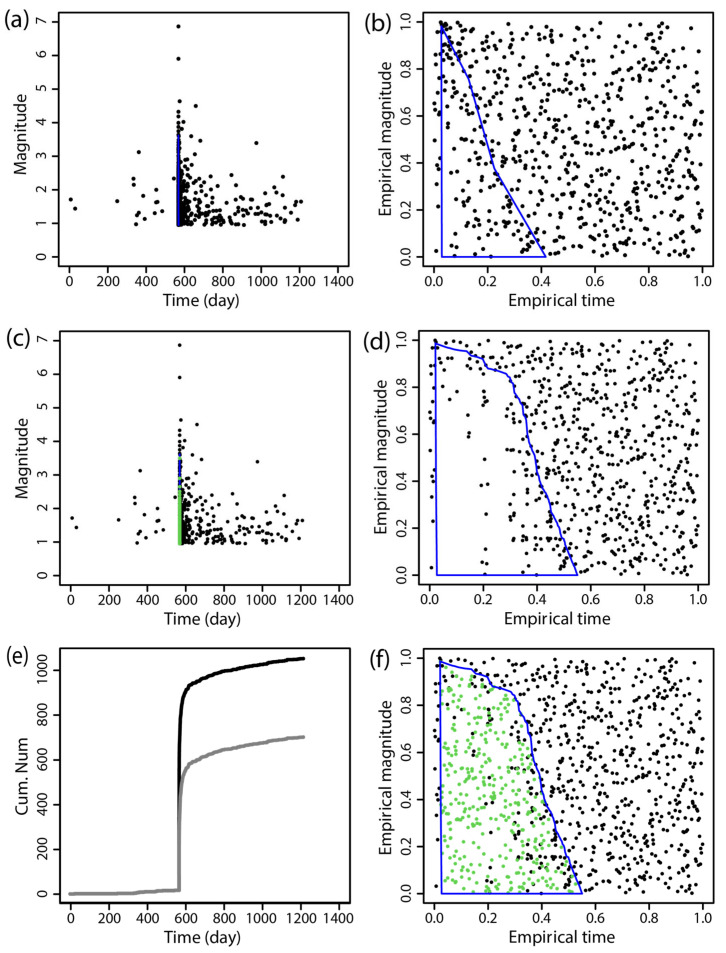
The supplementary results of the aftershock data of the 2013 *M*_S_6.6 Minxian earthquake obtained by using BEPIT. (**a**) shows the magnitude and occurrence time of actual seismic events and missing event ranges; (**b**) shows the empirical distribution of magnitude and the event occurrence time after the double-scale transformation technique (Step 1); (**c**) shows the magnitude and occurrence time of actual and supplementary seismic events (Step 5); (**d**) shows the empirical distribution of magnitude and the event occurrence time outside of the missing event range after the double-scale transformation technique (Steps 2 and 3); (**e**) shows the cumulative number of earthquakes in the original data set (gray curve) and the supplementary data set (black curve); and (**f**) shows the empirical distribution of the magnitude and the timing of actual and supplementary seismic events (Step 4). The blue polygon in (**a**,**b**,**d**,**f**) is the region where the missing event is located, and its corresponding mapping and the green dots in (**c**,**f**) are the supplementary events. The *x*-axis and *y*-axis in (**b**,**d**,**f**) are the empirical time and empirical magnitude transformed from time and magnitude by BEPIT.

**Figure 4:**
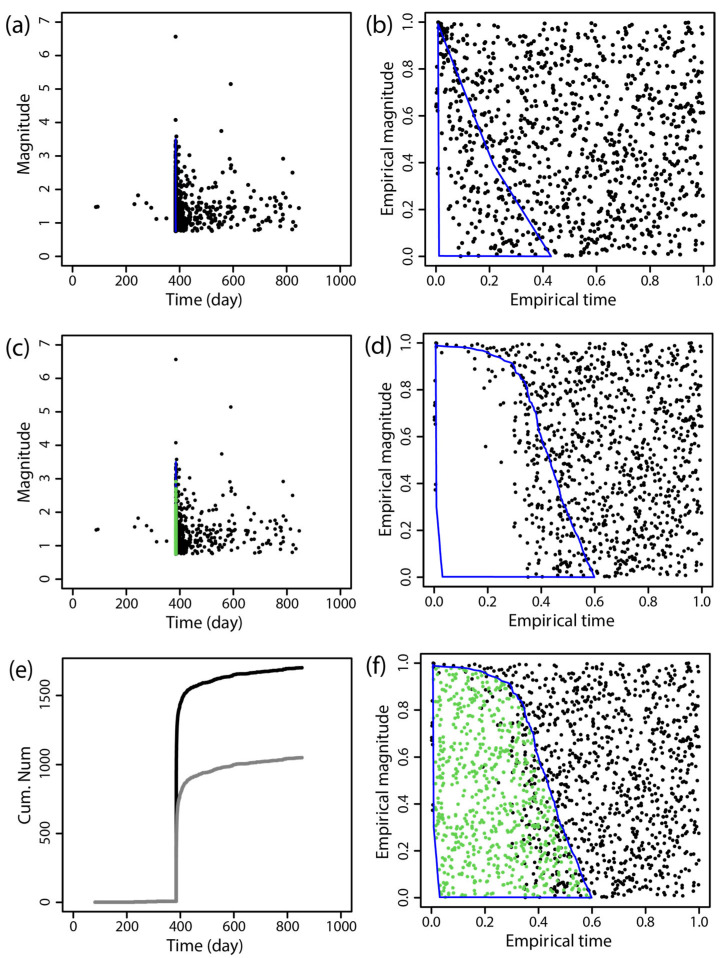
The supplementary results of aftershock data of the 2016 *M*_S_6.4 Menyuan earthquake obtained by using BEPIT. (**a**) shows the magnitude and occurrence time of actual seismic events and missing event ranges; (**b**) shows the empirical distribution of magnitude and the event occurrence time after the double-scale transformation technique (Step 1); (**c**) shows the magnitude and occurrence time of actual and supplementary seismic events (Step 5); (**d**) shows the empirical distribution of magnitude and the event occurrence time outside of the missing event range after the double-scale transformation technique (Steps 2 and 3); (**e**) shows the cumulative number of earthquakes in the original data set (gray curve) and the supplementary data set (black curve); and (**f**) shows the empirical distribution of the magnitude and the timing of actual and supplementary seismic events (Step 4). The blue polygon in (**a**,**b**,**d**,**f**) is the region where the missing event is located, and its corresponding mapping and the green dots in (**c**,**f**) are the supplementary events. The *x*-axis and *y*-axis in (**b**,**d**,**f**) are the empirical time and empirical magnitude transformed from time and magnitude by BEPIT.

**Figure 5:**
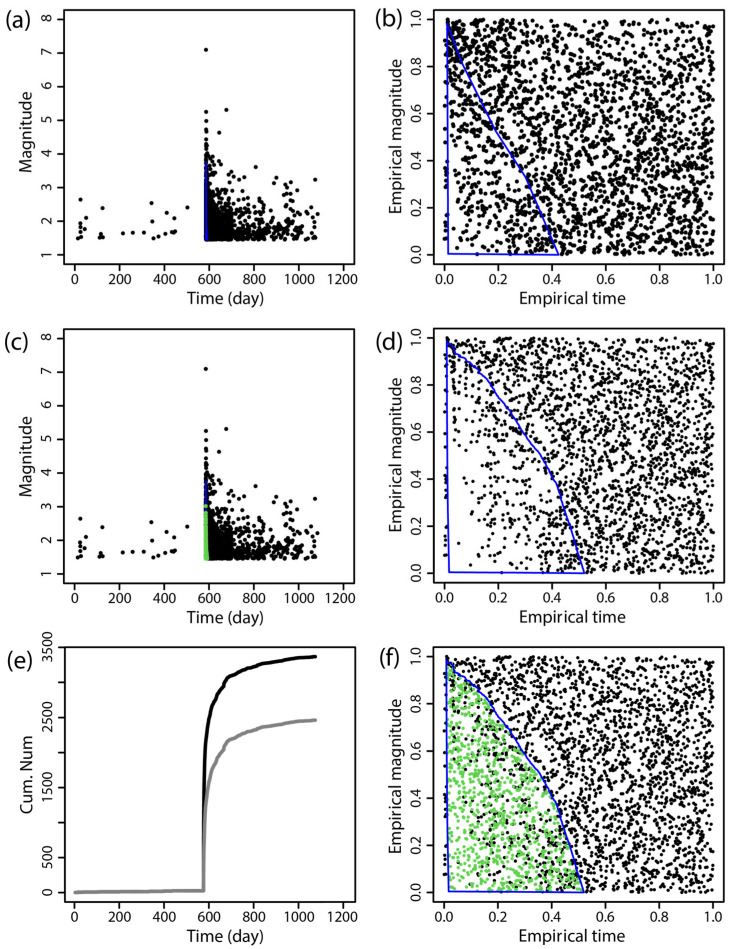
The supplementary results of aftershock data of the 2017 *M*_S_7.0 Jiuzhaigou earthquake obtained by using BEPIT. (**a**) shows the magnitude and occurrence time of actual seismic events and missing event ranges; (**b**) shows the empirical distribution of magnitude and the event occurrence time after the double-scale transformation technique (Step 1); (**c**) shows the magnitude and occurrence time of actual and supplementary seismic events (Step 5); (**d**) shows the empirical distribution of magnitude and the event occurrence time outside of the missing event range after the double-scale transformation technique (Steps 2 and 3); (**e**) shows the cumulative number of earthquakes in the original data set (gray curve) and the supplementary data set (black curve); and (**f**) shows the empirical distribution of the magnitude and timing of actual and supplementary seismic events (Step 4). The blue polygon in (**a**,**b**,**d**,**f**) is the region where the missing event is located, and its corresponding mapping and the green dots in (**c**,**f**) are the supplementary events. The *x*-axis and *y*-axis in (**b**,**d**,**f**) are the empirical time and empirical magnitude transformed from time and magnitude by BEPIT.

**Figure 6:**
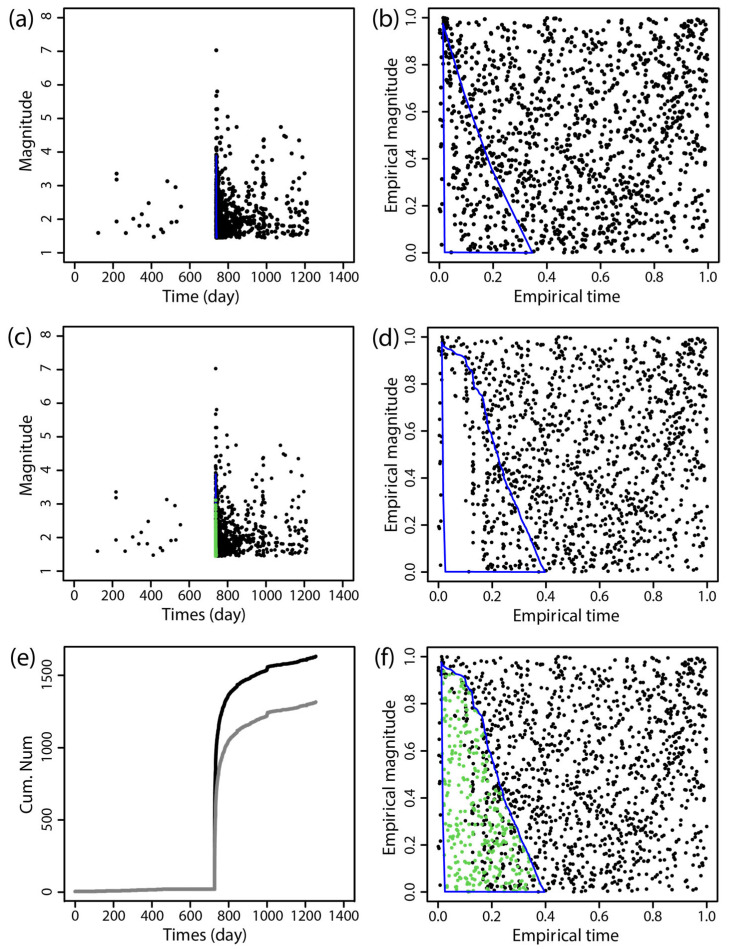
The supplementary results of aftershock data of the 2022 *M*_S_6.9 Menyuan earthquake obtained by using BEPIT. (**a**) shows the magnitude and occurrence time of actual seismic events and missing event ranges; (**b**) shows the empirical distribution of magnitude and the event occurrence time after the double-scale transformation technique (Step 1); (**c**) shows the magnitude and occurrence time of actual and supplementary seismic events (Step 5); (**d**) shows the empirical distribution of magnitude and the event occurrence time outside of the missing event range after the double-scale transformation technique (Steps 2 and 3); (**e**) shows the cumulative number of earthquakes in the original data set (gray curve) and the supplementary data set (black curve); and (**f**) shows the empirical distribution of the magnitude and timing of actual and supplementary seismic events (Step 4). The blue polygon in (**a**,**b**,**d**,**f**) is the region where the missing event is located, and its corresponding mapping and the green dots in (**c**,**f**) are the supplementary events. The *x*-axis and *y*-axis in (**b**,**d**,**f**) are the empirical time and empirical magnitude transformed from time and magnitude by BEPIT.

**Figure 7:**
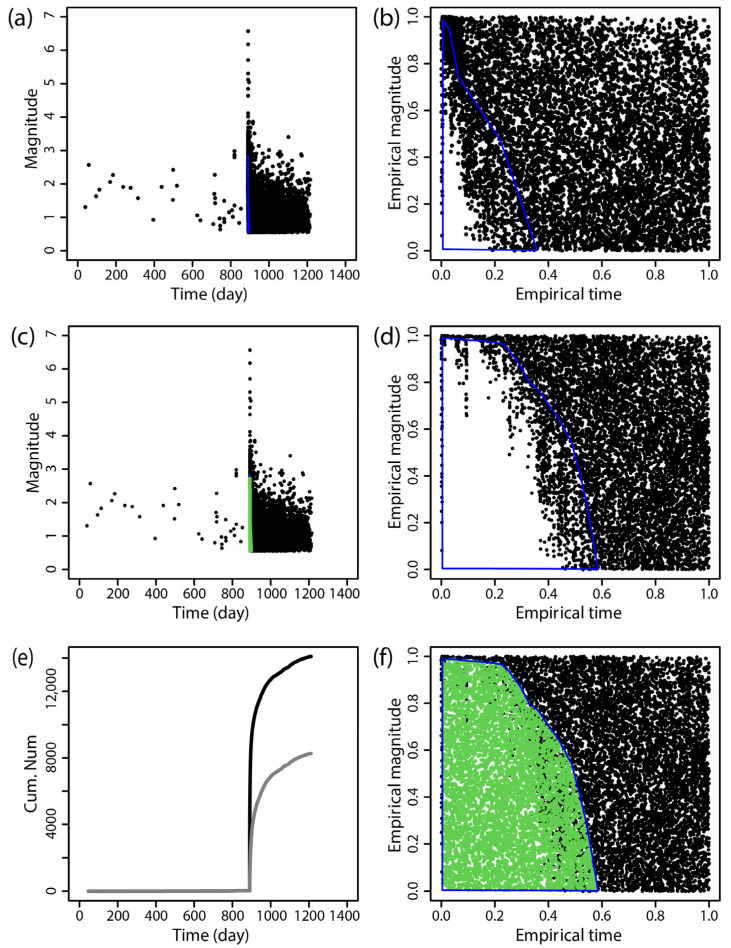
The supplementary results of aftershock data of the 2022 *M*_S_6.0 Maerkang earthquake obtained by using BEPIT. (**a**) shows the magnitude and occurrence time of actual seismic events and missing event ranges; (**b**) shows the empirical distribution of magnitude and the event occurrence time after the double-scale transformation technique (Step 1); (**c**) shows the magnitude and occurrence time of actual and supplementary seismic events (Step 5); (**d**) shows the empirical distribution of magnitude and the event occurrence time outside the missing event range after the double-scale transformation technique (Steps 2 and 3); (**e**) shows the cumulative number of earthquakes in the original data set (gray curve) and the supplementary data set (black curve); and (**f**) shows the empirical distribution of the magnitude and timing of actual and supplementary seismic events (Step 4). The blue polygon in (**a**,**b**,**d**,**f**) is the region where the missing event is located, and its corresponding mapping and the green dots in (**c**,**f**) are the supplementary events. The *x*-axis and *y*-axis in (**b**,**d**,**f**) are the empirical time and empirical magnitude transformed from time and magnitude by BEPIT.

The BEPIT method was applied to supplement missing earthquake data immediately following the main shock. Figure 2, Figure 3, Figure 4, Figure 5, Figure 6 and Figure 7 illustrate the results of this supplementation for the early aftershock data of six sequences. The findings show a significant increase in the number of recorded seismic events shortly after the main earthquake, highlighting the effectiveness of this supplementation technique. The enhanced seismic catalog provides a more comprehensive primary data set for subsequent research, enabling the derivation of more stable parameters.

**Figure 8:**
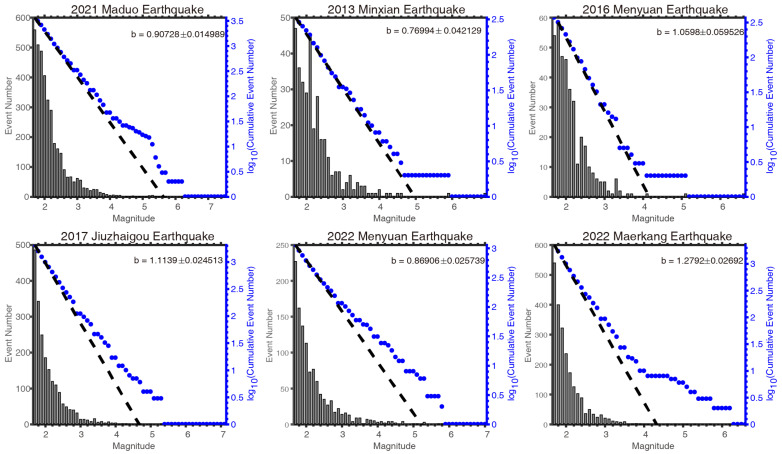
The *b* values of the six seismic sequences. The histogram represents the magnitude–frequency distribution of the sequence, the blue dots represent the magnitude–frequency distribution under the logarithmic relationship, and the black dashed lines represent the results of fitting using the G-R.

The ETAS model parameters for the six seismic sequences are estimated using maximum likelihood estimation based on a relatively complete catalog. To facilitate comparison and analysis across the six sequences, a unified *M*_C_ = *M*_L_1.7 is selected for calculation. Table 2 presents the estimated model parameters. Additionally, the maximum likelihood method is employed to estimate the *b* values of each seismic sequence (Figure 8).

An analysis of six earthquake sequences in the northeastern Tibetan Plateau revealed that although these sequences occur within the same tectonic region and share some general characteristics, they also display distinct parameter differences. As shown in Table 2, the *μ* values for all six sequences are low, indicating generally low seismic activity in these areas, with few earthquakes occurring before the main shock, particularly in sequences 2, 4, 5, and 6. Observational records confirm that no significant earthquakes occurred near the epicenters in the months preceding the 2013 Minxian *M*_S_6.6, 2017 Jiuzhaigou *M*_S_7.0, 2022 Menyuan *M*_S_6.9, and 2022 Maerkang *M*_S_6.0 earthquakes, making it challenging to assess earthquake hazards through foreshock activity. Sequence 3, however, has a relatively higher *μ* value, suggesting that background seismic activity in this region was higher than in the other sequences.

Parameters *A* and α collectively reflect aftershock productivity [52,53]. Specifically, parameter *A* represents the number of aftershocks triggered by an earthquake of magnitude *M*_C_, while parameter *α* indicates the relationship between an earthquake’s magnitude and its potential to generate aftershocks [7,54]. A high *α* suggests that a significant portion of aftershocks are triggered by larger earthquakes, primarily the mainshock. In sequences 2, 3, and 5, the high *α* value indicates a lower proportion of high-order aftershocks, with most aftershocks primarily being triggered by the mainshock.

The statistical decay of aftershocks is governed by parameters *c* and *p*. Sequence 5 exhibits the smallest *c* value, indicating a shorter time for the aftershock frequency to peak. A larger *p* value suggests a faster decay rate. Utzu et al. [55], in their analysis of over 200 global seismic sequences, reported an average *p* of 1.1. In the six sequences presented in Table 2, sequences 1–4 display a *p* value exceeding this average, suggesting that aftershocks decay rapidly following large earthquakes, potentially reducing the relative damage from aftershock sequences. The *b* value reveals that aside from sequence 6, overall, the *b* values are lower, reflecting regional seismic statistical characteristics and indicating high stress levels in the northeastern Tibetan Plateau [56,57,58].

## 4. Discussion

### 4.1. ETAS Parameter

The effect of different cutoff magnitudes on seismic sequence parameters is analyzed using the 2017 Jiuzhaigou *M*_S_7.0 earthquake sequence as a case study, and the results are presented in Table 3. By varying the cutoff magnitudes to *M*_L_ 1.7, 1.8, and 1.9, parameters *μ*, *A*, and *c* gradually decrease while *α* increases, and *p* remains largely unchanged. These results align with the typical parameter variation characteristics of the ETAS model, indicating that the supplementary catalog is relatively stable. Furthermore, the *b* value decreases slightly as the cutoff value increases, remaining within the margin of error, which suggests the stability of *b* and the suitability of the chosen cutoff magnitudes. Therefore, when examining seismic sequence parameters, the selection of the cutoff magnitude should be carefully considered.

Among the six seismic sequences analyzed in this study, five are mainshock–aftershock types, with sequence 6 exhibiting a swarm-type clustering pattern. As shown in Table 2, the ETAS model parameters vary significantly across these sequence types. The swarm-type sequence (sequence 6) demonstrates a higher *b* value, lower *α* value, and reduced *c* value and *p* value compared to the five mainshock–aftershock sequences. These parameter differences suggest that swarm sequences may feature relatively frequent aftershocks, slower attenuation rates post-event, and a heightened capacity to trigger second-order aftershocks. After a major earthquake, determining the sequence type is essential for assessing post-earthquake trends. The parameter characteristics observed here provide valuable insights for post-earthquake sequence classification and trend assessment.

The six seismic sequences examined in this study are situated on the Tibetan Plateau and represent continental intraplate earthquakes. Compared with subduction zone earthquakes, such as those in Japan, these intraplate sequences exhibit higher *p* and lower *c* values [7,59,60]. Consequently, the *p* and *c* values can also serve as indicators of tectonic environments.

### 4.2. Implication on Earthquake Forecasting

To investigate this further, we analyzed the probability of each mainshock being a background event [36], and the findings are presented in Table 2. With the exception of the 2022 *M*_s_6.0 Maerkang earthquake, most of the primary earthquakes exhibit a high probability of being background events, indicating limited foreshock activity and suggesting that these main events were not foreshock-triggered. Indeed, none of these significant earthquakes were preceded by foreshocks. Furthermore, due to the sparse earthquake activity before the main events, it is challenging to observe the long-term decline in the *b* value that has been observed before some major earthquakes, such as the Wenchuan earthquake, the Tohoku earthquake in Japan, and the Sumatra earthquake [48,49,61,62]. This scarcity of pre-event seismicity complicates earthquake risk assessment based solely on precursor activity.

Due to the low incidence of background earthquakes in the northeastern Tibetan Plateau, seismicity-based prediction methods are challenging to apply in this area. In addition to statistical seismicity modeling, other effective earthquake prediction approaches rely on non-seismic precursors. Currently, geomagnetic field variations and underground fluid anomalies serve as relatively mature earthquake precursors in this region. Approximately six months prior to the 2022 Menyuan *M*_S_6.9 earthquake, eight underground fluid anomalies were observed, primarily within a 200 km radius of the epicenter [63]. Approximately two months before the Menyuan *M*_S_ 6.9 earthquake, anomalously high geomagnetic polarization values exceeding the threshold were detected [64]. These geomagnetic observations, combined with recorded underground fluid anomalies, facilitated a successful prediction of the event [65]. Thus, earthquake forecasting in the northeastern Tibetan Plateau might be enhanced by integrating precursor-based approaches [66,67,68]. In addition to advancing seismic network capabilities, establishing dedicated precursor observation stations should be prioritized to improve predictive accuracy in this region.

The probability of the mainshock in the 2022 *M*_s_6.0 Maerkang earthquake sequence being a background event is nearly zero, indicating that it was triggered by preceding foreshock activity. Consistent with previous findings, this sequence exhibited a concentration of foreshocks across multiple faults prior to the mainshock, likely attributable to the region’s complex seismogenic environment. The *M*_s_6.0 Maerkang earthquake, the largest event in this swarm, was preceded by an *M*_s_5.8 foreshock and was followed by an *M*_s_5.2 event, resulting in three earthquakes being above *M*_s_5.0. This earthquake swarm occurred at the intersection of the Songgang and Longriba faults within the Bayan Har block, an area characterized by a complex epicentral structure. Studies on the precise location of the sequences suggest that the Maerkang swarm is influenced by multiple parallel and small-scale conjugate faults in proximity to the epicenter [69,70,71]. These three *M*_s_5.0+ earthquakes took place on distinct fault sections, forming a swarm driven by multi-fault planes [72]. Compared to single-fault systems, such complex, multi-fault structures can yield more intricate earthquake sequences. Thus, in regions with similarly complex fault systems, the possibility of subsequent moderate to large earthquakes following an initial significant event should be anticipated.

### 4.3. Constraints and Challenges

As this research progressed, constraints in the application of certain methods also became apparent. One notable limitation pertains to the Gutenberg–Richter (G-R) law. Beyond the requirement that earthquakes used for a *b* value analysis must have magnitudes equal to or greater than the completeness magnitude, the G-R law may not hold for large-magnitude earthquakes, as illustrated in Figure 8. To address this issue, some studies have introduced derivative forms of the G-R law based on statistical methods, but their practical application remains limited [73]. From the perspective of physical properties, the statistical analysis of seismic energy has been shown to yield more accurate fits and robust results [74,75].

Another limitation involves the determination of the missing event range in the BEPTI algorithm. In Step 2 of BEPTI, the time–magnitude range for missing events, SS, must be manually estimated. This range should encompass the entire region where earthquakes are missing and extend beyond it. However, the manual estimation of this range introduces subjectivity, which may affect the results, as discussed by Zhuang et al. [29]. While it has been suggested that the selection of this range is not highly sensitive to the final outcomes, the development of an algorithm for the objective determination of missing earthquakes is necessary to improve the method’s reliability and objectivity.

## 5. Conclusions

This study examined six *M*_S_ ≥ 6.0 earthquake sequences that have occurred in the northeastern Tibetan Plateau since 2009. Using the BEPIT method, missing earthquakes from the short-term period following major events were supplemented, resulting in a more comprehensive seismic sequence catalog. The findings confirm that BEPIT is a highly efficient method suitable for supplementing any earthquake sequence initially affected by limited early monitoring, facilitating the assessment of missing data impacts and the correction of related biases. A subsequent analysis of the ETAS model parameters for these six sequences yielded the following conclusions.

The *μ* values for the six seismic sequences in the northeastern Tibetan Plateau are uniformly low, suggesting a generally low background seismicity rate with few foreshocks preceding major earthquakes. This underscores the challenges in forecasting earthquakes in this region solely through seismic activity analysis and foreshock observations, providing critical insights for the strategic planning of earthquake prediction efforts. The findings of this research provide valuable reference points for designing seismic monitoring strategies prior to large earthquakes and for analyzing changes in regional seismic sequence parameters. Additionally, they can contribute to improving the scientific accuracy of early assessments of post-earthquake trends.

The parameters of the six earthquake sequences in the northeastern Qinghai–Tibet Plateau examined in this study differ from those observed in previous earthquake cases within mainland China and subduction zones [59,60,76]. These differences arise from variations in regional stress conditions, seismogenic structures, and crustal medium properties.

It is important to note that the study area experienced a limited number of strong earthquakes after the upgrade of the seismic network, resulting in the selection of only six earthquake sequences for analysis. Consequently, the findings of this study may capture only a subset of statistical characteristics. Further research incorporating a larger set of earthquake cases is needed for a more comprehensive and in-depth analysis.

## Figures and Tables

**Table 1:** The *M*_C_ and the supplementary results of six seismic sequences.

Time (dd-mm-yyyy)	Region	*M*_S_	*M*c Before Supplementing	*M*c After Supplementing
22 May 2021	Maduo	7.4	2.9	1.5
22 July 2013	Minxian	6.6	2.4	1.0
22 January 2016	Menyuan	6.4	1.5	0.8
8 August 2017	Jiuzhaigou	7.0	2.1	1.5
8 January 2022	Menyuan	6.9	2.0	1.5
10 June 2022	Maerkang	6.0	2.0	0.6

**Table 2:** ETAS model parameter, *b* values and background earthquake probability of main earthquake of six seismic sequence.

Index	Seismic Sequence			Sequence Type	Fault Type	*μ*	*A*	*α*	*c*	*p*	*b*	Background Earthquake Probability of Main Earthquake
	Time (dd-mm-yyyy)	Region	*M*_S_									
1	22 May 2021	Maduo	7.4	M-A	strike-slip	0.0182	0.2711	1.0767	0.0823	1.1842	0.9073 ± 0.015	0.7625
2	22 July 2013	Minxian	6.6	M-A	strike-slip and thrust	0.0001	0.0032	2.2439	0.0121	1.1032	0.7700 ± 0.042	0.7522
3	22 January 2016	Menyuan	6.4	M-A	thrust	0.0328	0.0005	2.6892	0.1087	1.2776	1.0598 ± 0.060	1.0000
4	8 August 2017	Jiuzhaigou	7.0	M-A	strike-slip	0.0002	0.0738	1.9214	0.0291	1.1547	1.1139 ± 0.025	0.2853
5	8 January 2022	Menyuan	6.9	M-A	strike-slip	0.0022	0.0173	2.2601	0.0051	1.0388	0.8691 ± 0.026	0.9241
6	10 June 2022	Maerkang	6.0	S	strike-slip	<0.0001	0.2689	1.7713	0.0071	1.0576	1.2792 ± 0.027	<0.001

M-A: mainshock–aftershock type; S: earthquake swarm type.

**Table 3:** ETAS model parameter and *b* values of 2017 Jiuzhaigou *M*_S_7.0 earthquake sequence at different cutoff magnitudes.

Cutoff Magnitude	*μ*	*A*	*α*	*c*	*p*	*b*
*M*_L_1.7	0.000192	0.073782	1.921365	0.029117	1.154718	1.1139 ± 0.025
*M*_L_1.8	0.000293	0.020183	2.230346	0.016399	1.178645	1.0088 ± 0.032
*M*_L_1.9	0.000255	0.018494	2.257887	0.009518	1.168921	1.0089 ± 0.035

## Data Availability

The catalog in this study was provided by the China Earthquake Networks Center from 2009 to June 2023.
